# Impact of early hyperoxia on outcomes during neonatal and pediatric veno-arterial extracorporeal life support★

**DOI:** 10.1051/ject/2025057

**Published:** 2026-03-13

**Authors:** Ashish Saini, Rebecca Shamah, Joshua Qian, Kasey Keane-Lerner, Paola Rodriguez Morales, Pranay Nayi, Adithi Shyam, Joel Davis, Mohan John, Heather Viamonte, Assad G Beshish

**Affiliations:** 1 Department of Pediatrics, Division of Cardiology, Emory University School of Medicine, Children’s Healthcare of Atlanta Atlanta GA USA; 2 Emory University School of Medicine Atlanta GA USA; 3 Physician Assistant, Children’s Healthcare of Atlanta Atlanta GA USA; 4 Research Coordinator, Children’s Healthcare of Atlanta Atlanta GA USA; 5 Advance Technology Coordinator, ECMO and Advanced Technologies, Children’s Healthcare of Atlanta Atlanta GA USA; 6 Department of Surgery, Division of Cardiothoracic Surgery, Emory University School of Medicine, Children’s Healthcare of Atlanta Atlanta GA USA

**Keywords:** Hyperoxia, ECLS, Mortality, ECLS Complications, Functional Status Scale

## Abstract

*Background:* Hyperoxia induces oxidative stress and can exacerbate inflammatory response to Veno-Arterial Extracorporeal Life Support (VA–ECLS). This study aimed to evaluate the association between hyperoxia during VA–ECLS and morbidity, complications, and in-hospital mortality. *Methods:* This study included pediatric patients who received VA–ECLS between 2014 and 2019. Hyperoxia severity was categorized as mild (PaO_2_: 101–200 mmHg), moderate (PaO_2_: 201–300 mmHg), and severe (PaO_2_ > 300 mmHg. The primary outcome was all-cause in-hospital mortality. Secondary outcomes included a composite measure of cardiovascular or renal complications, AKI, and change in Functional Status. *Results:* Among 229 patients supported on VA–ECLS runs, 73.4% involved neonates. Median age and weight of the entire cohort were 2.5 months (IQR 0.3, 19.0), and 4.4 kg (IQR 3.2, 10.7), respectively. Cardiac indications accounted for 48.9% of cases. Hyperoxia occurred in 79% of patients and was more common in those requiring ECLS for cardiac indications. The overall in-hospital mortality rate was 45%, increasing to 64% in the severe hyperoxia cohort (*p* = 0.23). Severe hyperoxia was significantly associated with the composite outcome of cardiovascular or renal complications but not in-hospital mortality in multivariable analysis. No association was found between hyperoxia, AKI, and adverse functional outcomes. Conclusions: Standardized PaO_2_ targets to minimize hyperoxia may improve outcomes for patients supported on VA–ECLS.

**Abbreviations**
ABG: Arterial Blood Gas
AKI: Acute Kidney Injury
E–CPR: Extracorporeal Cardiopulmonary Resuscitation
ELSO: Extracorporeal Life Support Organization
FSS Score: Functional Status Scale Score
PaO_2_
: Partial Pressure of Oxygen
VA–ECLS: Veno-Arterial Extracorporeal Life Support

## Introduction

Hyperoxia has been associated with adverse clinical outcomes and increased mortality in various scenarios, including traumatic brain injury, perinatal asphyxia, critical illness, and post-resuscitation states [1–5]. Its deleterious effects are attributable to cellular dysfunction, inflammation, and necrosis due to enhanced production of reactive oxygen species [6]. Veno-arterial (VA) extracorporeal life support (ECLS) provides circulatory support by removing blood from the venous system and returning highly oxygenated blood to the arterial circulation. VA–ELCS is commonly used to support neonatal and pediatric patients with severe cardiopulmonary failure and after cardiac arrest [7]. However, ECLS itself induces systemic inflammation and oxidative stress similar to hyperoxia [8]. Therefore, exposure to hyperoxia during ECLS can further exacerbate tissue injury and inflammation, leading to worsened outcomes in patients supported by ECLS.

Previous studies have reported an association between hyperoxia during ECLS or cardiopulmonary bypass with increased mortality in pediatric patients [9–11]. However, it remains unclear whether the deleterious effect of hyperoxia occurs beyond a specific threshold or follows a dose-dependent effect, with evidence supporting both possibilities [4, 10]. Furthermore, the definition of hyperoxia varies across studies. This study aims to expand on these findings by focusing exclusively on pediatric patients supported by VA–ECMO across all indications to determine the association between hyperoxia and poor outcomes. Furthermore, hyperoxia was stratified into three distinct levels of severity (mild, moderate, and severe) to investigate a potential threshold for predicting adverse outcomes.

## Materials and methods

This single-center retrospective cohort study included all neonatal and pediatric patients who required VA–ECLS between January 1st, 2014, and December 31st, 2019, at Children’s Healthcare of Atlanta (CHOA), a free-standing, university-affiliated quaternary children’s hospital. Eligible patient encounters were identified through an internal ECLS database. Preterm neonates (gestational age < 37 weeks) as well as patients less than 2 kg at time of ECMO initiation were excluded. The primary objective was to assess whether hyperoxia during VA–ECLS is associated with increased in-hospital mortality. The secondary objective was to determine whether hyperoxia is associated with higher odds of complications and morbidity using standardized and generalizable definitions. The study was approved by the CHOA Institutional Review Board (IRB# 00001239), with informed consent waived.

## Data and definitions

All consecutive patients who required VA–ECLS during their index hospitalization in the neonatal, pediatric, and cardiac intensive care units were included in the study. For patients with multiple ECLS courses, only the first ECLS run was included. Demographic data, clinical characteristics, and ECLS-related variables were collected. The indication for ECLS was categorized as cardiac, extracorporeal cardiopulmonary resuscitation (E–CPR), or respiratory based on the extracorporeal life support organization (ELSO) categorization. All arterial blood gas (ABG) measurements obtained during the first 48 h of ECLS initiation were reviewed for the partial pressure of oxygen (PaO_2_). Normoxia was defined as PaO_2_ of ≤ 100 mmHg. Hyperoxia was categorized based on the single highest PaO_2_ value as mild (PaO_2_: 101–200 mmHg), moderate (PaO_2_: 201–300 mmHg), and severe (PaO_2_ > 300 mmHg**).** These categories were chosen to create clinically relevant strata based on prior studies [9, 10]. ECLS complications based on definitions from the ELSO registry were documented, including cardiovascular, hematologic, mechanical, renal, neurologic, metabolic, and infectious complications [12]. Additionally, the incidence of Acute kidney injury (AKI) was recorded using the KDIGO scoring criteria [13]. The primary outcome was all-cause in-hospital ECLS mortality. Secondary outcomes included a composite measure of cardiovascular or renal complications (per ELSO definitions), incidence of AKI, and change in Functional Status Scale (FSS) score.

## Functional Status Scale (FSS)

The FSS consists of 6 main domains: mental status, sensory, communications, motor function, feeding, and respiratory. Each domain is scored from 1 (normal) to 5 (severe dysfunction), with a total FSS score range of 6–30 [14]. Baseline and discharge FSS scores were determined retrospectively from clinical documentation, blinded to hyperoxia status. Newborns without a stable baseline function were assigned an FSS score of 6. This applied to all infants ≤ 2 days old at admission and those transferred from another facility between 3 to 6 days of age [15]. New morbidity was defined as an increase in the total FSS score of ≥ 3 points, while an unfavorable functional outcome was defined as an increase of ≥ 5 points [16].

## Clinical management

For patients < 40 kg, ECLS circuits were blood primed with packed red blood cells, 25% albumin, sodium-bicarbonate, calcium-gluconate, and heparin. The initial ABG was obtained at the clinical team’s discretion, typically 30 min after ECLS cannulation, followed by hourly ABGs for the first 3 h. Subsequent ABGs were obtained every 3–6 h and 30 min after any adjustment in ECLS support. Our center does not follow a standardized protocol for PaO_2_ targets, and the variations described reflect standard clinical care. Typical pH and PaCO_2_ goals at our institution are 7.35–7.45 and 35–45 mmHg, respectively. While these are general goals, there was no standardized protocol or timeline to achieve them, and management was at the discretion of the clinical team.

## Statistical analysis

Continuous variables were reported as median with interquartile ranges (IQR) and compared using Kruskal Wallis test. Categorical variables were expressed as numbers and percentages and compared using a chi-square test. The impact of hyperoxia on primary and secondary outcome variables was assessed using univariable and multivariable logistic regression, adjusting for BSA, age group, and indication for ECLS. A subgroup analysis was performed for neonates to investigate differential effects in this population. The results were reported as unadjusted and adjusted odds ratios (OR) with corresponding 95% confidence intervals (CI). Statistical analysis was conducted using SAS software, version 9.4M8 (Cary, NC: SAS Institute Inc; 2023), with a significance level set at *p* < 0.05.

## Results

A total of 229 patients were supported on VA–ECLS during the study period, with neonates comprising 73.4% of the cohort. The median age and weight were 2.5 months (IQR 0.3, 19), and 4.4 kg (IQR 3.2, 10.7), respectively. Cardiac indications accounted for 48.9% of the ECLS runs. The median time from admission to ECLS initiation was 78.5 h (IQR 14, 356), and the median duration of ECLS support was 111.5 h (IQR 65.5, 184.5). The overall in-hospital mortality rate was 45% and Stage II or higher AKI was 75.8%. Additional demographic and clinical variables are presented in Table 1.

**Table 1:** Patient demographics and clinical characteristics of entire VA-ECLS cohort.

Variables	Total cohort (*n* = 229)
Age (months)	2.5 (0.3, 19.0)
Age group	
Neonates	168 (73.4%)
Pediatrics	61 (26.6%)
Weight (kg)	4.4 (3.2, 10.7)
BSA (m^2^)	0.3 (0.2, 0.5)
Race	
Black	104 (45.4%)
White	87 (38.0%)
Hispanic	26 (11.4%)
Other	12 (5.2%)
Sex	
Female	115 (50.2%)
Male	114 (49.8%)
ECLS indication	
Cardiac	112 (48.9%)
ECPR	65 (28.4%)
Pulmonary	52 (22.7%)
Time from admission to ECLS initiation (hours)	78.5 (14.0, 356.0)
Duration of ECLS run (hours)	111.5 (65.5, 184.5)
ECLS complications:	
Cardiovascular	87 (45.8%)
Hemorrhagic	86 (45.3%)
Mechanical	75 (39.5%)
Renal	103 (54.2%)
Neurologic	46 (24.2%)
Metabolic	29 (15.3%)
Infection	9 (4.7%)
AKI Stage II or III	160 (75.8%)
Mortality	103 (45%)

Results Depicted in *n* (%), and Median (Interquartile Range/IQR). AKI: Acute Kidney Injury, BSA: Body Surface Area, ECLS: Extracorporeal Life Support; VA: Veno-Arterial.

**Figure 1:**
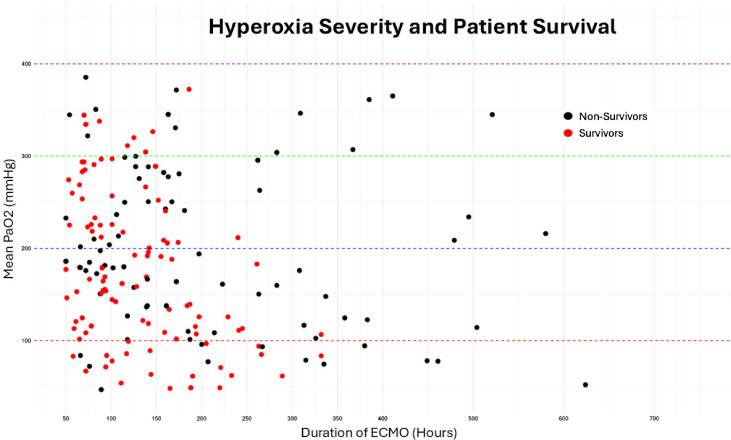
Scatter plot of patient survival by severity of hyperoxia. Each dot represents an individual patient, with survivors in red and non-survivors in black. The colored dashed lines represent the PaO_2_ threshold levels of 100 (red), 200 (blue), and 300 (green).

A total of 181 patients (79%) experienced hyperoxia, with 87 patients (37.9%) exposed to moderate to severe hyperoxia. Table 2 summarizes demographic characteristics and clinical data stratified by hyperoxia severity. Patients with PaO_2_ between 201 and 300 mmHg were older, with a median age of 7.9 months (IQR 0.5, 50.7) and a median weight of 7.4 Kg (IQR 3.5, 19.2). Moderate to severe hyperoxia was significantly associated with a cardiac indication for ECLS (*p* < 0.01). Patients with severe hyperoxia (PaO_2_ > 300 mmHg) had the highest in-hospital mortality rate (16/25, 64%) and had the highest incidence of cardiovascular (60%), renal (68%) complications, and stage II or higher AKI (95.2%) (Table 2). The relationship between PaO_2_ and patient survival is depicted in Figure 1, which demonstrates a proportionately smaller number of survivors among patients exposed to severe hyperoxia.

**Table 2:** Patient demographics and clinical characteristics stratified by hyperoxia severity.

Variables	Normoxia PaO_2_ ≤ 100 mmHg (*n* = 48)	Mild hyperoxia PaO_2_ 101–200 mmHg (*n* = 94)	Moderate hyperoxia PaO_2_ 201–300 mmHg (*n* = 62)	Severe hyperoxia PaO_2_ > 300 mmHg (*n* = 25)	*p*-value
Age (months)	0.5 (0.1, 2.9)	2.5 (0.3, 18.3)	7.9 (0.5, 50.7)	3.2 (0.4, 9.6)	**0.001**
Age group					**0.01**
Neonates	42 (87.5%)	69 (73.4%)	37 (59.7%)	20 (80.0%)	
Pediatrics	6 (12.5%)	25 (26.6%)	25 (40.3%)	5 (20.0%)	
Weight (kg)	3.4 (2.9, 4.4)	4.3 (3.2, 11.4)	7.4 (3.5, 19.2)	4.4 (3.0, 8.1)	**0.001**
BSA (m^2^)	0.2 (0.2, 0.3)	0.2 (0.2, 0.5)	0.3 (0.2, 0.7)	0.3 (0.2, 0.4)	**0.007**
Race					0.2
Black	29 (60.4%)	37 (39.4%)	104 (45.4%)	104 (45.4%)	
White	3 (6.3%)	15 (16.0%)	87 (38.0%)	87 (38.0%)	
Hispanic	3 (6.3%)	6 (6.4%)	26 (11.4%)	26 (11.4%)	
Other	13 (27.1%)	36 (38.3%)	12 (5.2%)	12 (5.2%)	
Sex					0.7
Female	21 (43.8%)	47 (50.0%)	33 (53.2%)	14 (56.0%)	
Male	27 (56.3%)	47 (50.0%)	29 (46.8%)	11 (44.0%)	
ECLS Indication					**<0.001**
Cardiac	16 (33.3%)	44 (46.8%)	37 (59.7%)	15 (60.0%)	
ECPR	9 (18.8%)	28 (29.8%)	20 (32.3%)	8 (32.0%)	
Pulmonary	23 (47.9%)	22 (23.4%)	5 (8.1%)	2 (8.0%)	
Time from admission to ECLS Initiation	105.0 (25.0, 593.0)	49.0 (14.0, 353.0)	78.5 (5.5, 257.0)	111.5 (24.0, 313.0)	0.57
Duration of ECLS (hours)	118.0 (43.5, 248.0)	112.0 (65.0, 185.0)	101.0 (68.0, 158.0)	125.0 (72.0, 186.0)	0.68
ECLS Complications:					
Cardiovascular	18 (45.0%)	28 (37.8%)	26 (51.0%)	15 (60.0%)	0.21
Hemorrhagic	16 (40.0%)	33 (44.6%)	25 (49.0%)	12 (48.0%)	0.84
Mechanical	20 (50.0%)	32 (43.2%)	13 (25.5%)	10 (40.0%)	0.09
Renal	21 (52.5%)	39 (52.7%)	26 (51.0%)	17 (68.0%)	0.52
Neurologic	5 (12.5%)	21 (28.4%)	11 (21.6%)	9 (36.0%)	0.12
Metabolic	5 (12.5%)	11 (14.9%)	8 (15.7%)	5 (20.0%)	0.88
Infection	4 (10.0%)	3 (4.1%)	1 (2.0%)	1 (4.0%)	0.33
AKI Stage II or III	34 (75.6%)	63 (74.1%)	43 (71.7%)	20 (95.2%)	0.17
Mortality	19 (39.6%)	41 (43.6%)	27 (43.5%)	16 (64.0%)	0.23

Results depicted as *n* (%), median (Interquartile range). AKI: Acute Kidney Injury, BSA: Body Surface Area, ECLS: Extracorporeal Life Support; VA: Veno-Arterial.

In univariable analysis, severe hyperoxia compared to normoxia was associated with higher odds of in-hospital mortality (OR 2.7, 95% CI 1.0–7.4, *p* = 0.04) and the composite outcome of cardiovascular or renal complications (OR 4.3, 95% CI 0.9–20.7, *p* = 0.04). There was a trend towards a higher incidence of stage II or higher AKI, which did not reach statistical significance (OR 6.5, 95% CI 0.8–53.9, *p* = 0.06). In multivariable regression analysis adjusted for age, body surface area (BSA), and indication for VA–ECLS, severe hyperoxia remained significantly associated with the composite outcome of cardiovascular or renal complications (OR 4.6, 95% CI 0.9–23.0, *p* = 0.03). The association with in-hospital mortality trended towards but did not reach statistical significance (OR 2.5, 95% CI 0.9–7.4, *p* = 0.1) (Table 3).

**Table 3:** Outcomes of patients undergoing VA–ECLS using a univariable and multivariable regression analysis.

Variables	OR (95% CI)	*p*-value	aOR^a^ (95% CI)	Adjusted *p*-value
Mortality				
Mild vs Ref.	1.2 (0.6–2.3)	0.43	1.3 (0.6–2.8)	0.66
Moderate vs Ref.	1.2 (0.6–2.5)	0.47	1.3 (0.6–3.1)	0.70
Severe vs Ref.	2.7 (1.0–7.4)	**0.04**	2.5 (0.9–7.4)	0.10
Any Cardiovascular or Renal complication				
Mild vs Ref.	0.9 (0.6–2.3)	0.12	0.8 (0.4–2.4)	0.06
Moderate vs Ref.	0.8 (0.6–2.5)	0.12	0.9 (0.3–2.4)	0.23
Severe vs Ref.	4.3 (0.9–20.7)	**0.04**	4.6 (0.9–23.0)	0.03
Stage II/III AKI**				
Mild vs Ref.	0.9 (0.4–2.1)	0.16	0.9 (0.4–2.4)	0.19
Moderate vs Ref.	0.8 (0.3–1.9)	0.09	0.9 (0.3–2.4)	0.14
Severe vs Ref.	6.5 (0.8–53.9)	0.06	6.2 (0.7–53.5)	0.07

Reference group = Normoxia (PaO_2_ ≤ 100)**.** Mild Hyperoxia (PaO_2_ = 101–200 mmHg); Moderate Hyperoxia (PaO_2_ = 201–300 mmHg); Severe Hyperoxia (PaO_2_ > 300 mmHg)**.** **Stage II AKI: Stage 2: serum creatinine increase of 2–2.9 times baseline, or UO < 0.5 mL/kg/h for ≥ 12 h; Stage III AKI: serum creatinine increase ≥ 3 times baseline or ≥ 4 mg/dL, or UO < 0.3 mL/kg/h for ≥ 24 h or anuria ≥ 12 h, or need for renal replacement therapy**.** ^a^ Adjusted for age group, BSA, indication for ECLS.

Subset analysis in neonates showed similar results, with higher odds of in-hospital mortality (OR 2.7, 95% CI 0.9–8.1, *p* = 0.03) and cardiovascular or renal complications (OR 15.3, 95% CI 1.9–124.1, *p* = 0.01) in univariate analysis when comparing the severe hyperoxia group to those without hyperoxia. The association remained significant for cardiovascular or renal complications (OR 11.9, 95% CI 1.4–100.4, *p* = 0.03) in multivariable regression analysis. Severe hyperoxia was not associated with stage II or higher AKI, either in univariable (OR 11.3, 95% CI 0.6–221.9, *p* = 0.11) or multivariable analysis (OR 19.4, 95% CI 0.8–452.9, *p* = 0.07) (Table 4).

**Table 4:** Outcomes of patients undergoing VA–ECLS using a univariable and multivariable regression analysis amongst neonates (age ≤ 30 days) *n* = 168.

Variables	OR (95% CI)	*p*-Value	aOR^a^ (95% CI)	Adjusted *p*-value
Mortality				
Mild vs Ref.	0.9 (0.4–1.9)	0.40	0.9 (0.4–2.0)	0.39
Moderate vs Ref.	0.7 (0.3–1.7)	0.09	0.8 (0.3–1.9)	0.19
Severe vs Ref.	2.7 (0.9–8.1)	**0.03**	2.4 (0.7–8.1)	0.06
Any Cardiovascular or Renal complications				
Mild vs Ref.	1.3 (0.6–2.7)	0.06	1.2 (0.5–2.6)	0.09
Moderate vs Ref.	1.7 (0.7–4.1)	0.36	1.4 (0.5–3.7)	0.30
Severe vs Ref.	15.3 (1.9–124.1)	**0.01**	11.9 (1.4–100.4)	**0.03**
Stage II/III AKI**				
Mild vs Ref.	1.1 (0.4–2.7)	0.21	1.2 (0.4–3.2)	0.15
Moderate vs Ref.	1.1 (0.4–3.1)	0.25	1.3 (0.4–4.5)	0.27
Severe vs Ref.	11.3 (0.6–221.9)	0.11	19.4 (0.8–452.9)	0.07

Reference group = Normoxia (PaO_2_ ≤ 100)**.** Mild Hyperoxia (PaO_2_ = 101–200 mmHg); Moderate Hyperoxia (PaO_2_ = 201–300 mmHg); Severe Hyperoxia (PaO_2_ > 300 mmHg)**.** ** Stage II AKI: Stage 2: serum creatinine increase of 2–2.9 times baseline, or UO < 0.5 mL/kg/h for ≥ 12 h; Stage III AKI: serum creatinine increase ≥ 3 times baseline or ≥ 4 mg/dL, or UO < 0.3 mL/kg/h for ≥ 24 h or anuria ≥ 12 h, or need for renal replacement therapy**.** ^a^ Adjusted for age group, BSA, indication for ECLS.

**Table 5:** New morbidity and unfavorable functional outcome for survivors who required VA–ECLS stratified by hyperoxia.

ECLS group	PaO_2_ ≤ 100 mmHg (*n* = 29)	PaO_2_ 101–200 mmHg (*n* = 53)	PaO_2_ 201–300 mmHg (*n* = 35)	PaO_2_ > 300 mmHg (*n* = 9)	*p*-Value
New morbidity	6 (20.7%)	12 (22.2%)	10 (28.6%)	2 (22.2%)	0.88^2^
Unfavorable outcome	2 (6.9%)	5 (9.3%)	3 (8.6%)	0 (0.0%)	0.81^2^

Total scores are the sum of subscale scores, ranging from 6 to 30. New Morbidity: Change in FSS score by 3 points. Unfavorable Outcome: Change in FSS score by 5 points. FSS: Functional Status Scale; ECLS: Extracorporeal Life Support. FSS Subscale scores range from 1 to 5.

Among 126 survivors for whom functional status could be assessed, there was no statistically significant association between the severity of hyperoxia and adverse functional outcomes. The incidence of new morbidity and unfavorable outcomes was similar across all groups, *p* = 0.88 and *p* = 0.81, respectively (Table 5).

## Discussion

Our study examines the outcomes of patients supported by VA–ECLS stratified by the severity of hyperoxia exposure. Nearly half of the VA–ECLS runs were performed for a cardiac indication. The overall in-hospital mortality was 45% and Stage II or higher AKI was 75.8%. Hyperoxia was more common in patients requiring VA–ECLS for a cardiac indication. Patients in the moderate hyperoxia group (PaO_2_ = 201–300 mmHg) were older and, consequently, had a higher weight compared to the rest of the cohort. Patients in the severe hyperoxia group had the highest in-hospital mortality rate of 64% and had the highest incidence of cardiovascular and renal complications, and stage II or higher AKI. Severe hyperoxia was associated with increased odds of composite outcome of cardiovascular or renal complications. Additionally, while severe hyperoxia was associated with higher odds of in-hospital mortality in univariable analysis, this association trended towards but did not reach statistical significance in multivariable regression analysis.

Hyperoxia potentiates the production of reactive oxygen species, which leads to lipid peroxidation, protein damage, and cell death [17]. It has been linked to adverse outcomes in various clinical scenarios. Studies have reported higher mortality and worse neurological outcomes in patients with traumatic brain injury, and infants with hypoxic ischemic encephalopathy exposed to a PaO_2_ level greater than 200 mmHg [1, 3]. In the critical care setting, exposure to high PaO_2_ levels correlates with increased mortality in both adult and pediatric populations [18–20]. Additionally, after cardiac arrest or E–CPR, hyperoxia is associated with worse outcomes, with one study demonstrating a 24% increase in mortality per 100 mmHg increase in PaO_2_ [4, 5, 21].

Hyperoxia during ECLS has been linked to worse outcomes. In a study of 93 infants undergoing cardiac surgery, a PaO_2_ of > 193 mmHg on VA ECLS within the first 48 post-operative hours was associated with higher 30-day mortality in both unadjusted (OR: 16.6, 95%CI: 3.17–305, *p* = 0.001) and adjusted (OR: 9.79, 95% CI: 1.18–81, *p* = 0.03) regression analysis. Moreover, patients exposed to hyperoxia had a higher incidence of renal replacement therapy, with an overall in-hospital mortality rate of 49% [10]. In another multi-centric study including 484 patients on VA (86.7%) and veno-venous (VV) (13.3%) ECLS, hyperoxia defined as PaO_2_ > 200 mmHg during the first 48 h was associated with higher mortality (OR 1.03, 95% CI: 1.01–1.04). However, no significant differences in functional status scale or renal complications were observed. Notably, patients exposed to hyperoxia were more likely to have a cardiac indication for ECLS. The in-hospital mortality was 45% in this study [9]. Similar findings have been reported in adult patients undergoing VA ECLS [22, 23]. Comparable to prior studies, the overall in-hospital mortality in our study was 45% and patients with PaO_2_ > 200 mmHg were older and more likely to have a cardiac indication for ECLS. This observation likely reflects the more stringent PaO_2_ targets typically adopted by the neonatal intensive care units compared to pediatric or cardiac intensive care units. Although severe hyperoxia was noted to be associated with in-hospital mortality in univariable analysis, this association did not remain significant in multivariable regression analysis, likely due to the small number of patients in this group. However, severe hyperoxia was significantly associated with the composite outcome of cardiovascular or renal complications during ECLS. The association between severe hyperoxia and Stage II or higher AKI trended towards significance, suggesting a potential link that requires further investigation. Consistent with the prior study by Cashen et al. [9], no difference in the functional status at discharge was noted based on hyperoxia severity.

Our study adds to the growing body of literature on the impact of early hyperoxia on outcomes in patients undergoing VA–ECLS. The first 48 h after initiating VA-ECLS are often characterized by a profound systemic inflammatory response, which can be exacerbated by hyperoxia. This early phase of ECLS is therefore a period of significant vulnerability to hyperoxia mediated organ injury. Our findings suggest a potential threshold effect with PaO_2_ greater than 300 mmHg, associated with adverse outcomes. The duration of exposure above this threshold may also play a role, but quantifying this clinically remains challenging. Despite technological refinements, mortality among patients supported by VA–ECLS remains high. Identifying modifiable risk factors such as hyperoxia is crucial for improving outcomes. Mitigating exposure to hyperoxia remains an important target for intervention. In the absence of robust literature, PaO_2_ targets vary across centers and among providers. There is significant heterogeneity in the definition of hyperoxia and the thresholds at which its deleterious effects manifest. Therefore, a collaborative effort to standardize PaO_2_ targets during VA–ECLS and prospectively evaluate its impact on outcomes is warranted.

## Limitations

Our study is limited by its single-center retrospective design. The PaO_2_ targets on ECLS varied based on the provider’s discretion. Although PaO_2_ measurements were obtained at dedicated time intervals, it is not possible to distinguish the effect of prolonged hyperoxia exposure from those of acute spikes in PaO_2_ levels. Additionally, the sample size may be insufficient to fully assess the impact of hyperoxia on the outcomes. Importantly, many of these limitations can be addressed in a multicenter validation study, which our group is currently pursuing.

## Conclusion

Our study demonstrates an association between hyperoxia and adverse outcomes in VA–ECLS patients, particularly among neonates and those with cardiac indication. Severe hyperoxia was associated with increased cardiovascular and renal complications. Given the variability in PaO_2_ targets, standardized guidelines must be formulated and investigated to validate these findings and optimize oxygen management strategies during VA ECLS.

## Data Availability

Data is not available for this article to the public per the CHOA IRB regulations.
